# Synergistic and protective effect of atorvastatin and amygdalin against histopathological and biochemical alterations in Sprague-Dawley rats with experimental endometriosis

**DOI:** 10.1186/s13568-019-0760-2

**Published:** 2019-03-19

**Authors:** Fang Hu, Yichuan Hu, Fangxin Peng

**Affiliations:** 1 Department of Reproductive Medicine, Maternal and Child Health Hospital of HuBei Province, Wuhan, 430072 Hubei China; 2 Department of Anesthesiology, Wuhan Union Hospital, Tongji Medical College, Huazhong University of Science and Technology, Wuhan, 430072 Hubei China

**Keywords:** Atorvastatin, Amygdalin, Cytokines, Endometriosis, Rats

## Abstract

The aim of the present study was to evaluate the protective effects of combined atorvastatin and amygdalin in a rat model of endometriosis. Tumor necrosis factor-α (TNF-α), interleukin-6 (IL-6), matrix metalloproteinase-2 (MMP-2) and MMP-9 levels in the peritoneal fluid were determined. The expression of TNF-α, IL-6, MMP-2, and MMP-9 mRNA, and the levels of lipid peroxidation, reduced glutathione (GSH), superoxide dismutase (SOD), catalase, and glutathione peroxidase (Gpx) were measured. Histopathological analysis was also conducted. The results showed that peritoneal TNF-α, IL-6, MMP-2, and MMP-9 levels were reduced by > 50%, and mRNA expression was decreased. Lipid peroxidation was considerably reduced, while GSH, SOD, Gpx, and catalase levels increased by > 40%. Reductions in leukocyte infiltration and fibrosis following treatment were also observed. Thus, our study suggested that combined treatment consisting of atorvastatin and amygdalin attenuates endometriosis. A detailed investigation of molecular mechanism of atorvastatin and amygdalin in endometriosis is needed.

## Introduction

Endometriosis is a gynecological disease of the endometrium, in which the endometrial tissue of the uterine lining grows outside the uterus (do Amaral et al. 2009), most commonly on the Fallopian tubes, ovaries and tissues around the ovaries and uterus (Sharpe-Timms 2002). Infertility and pelvic pain are the primary symptoms of endometriosis (Bulletti et al. 2010). Bulun (2009) reported that one in every seven women suffers from endometriosis, which causes infertility in 30–50% of cases.

The lesions in endometriosis require cell adhesion, migration, and proliferation for development (Van Langendonckt et al. 2002; Giudice and Kao 2004). Elevated levels of matrix metalloproteinase-2 (MMP2) and MMP-9 have also been reported (Chung et al. 2001; Osteen et al. 2003). The treatment of endometriosis includes hormone therapy, such as androgen and gonadotropin-releasing hormone, and the use of non-steroidal anti-inflammatory drugs for pain relief (Garai et al. 2006). However, hormone therapy can result in side effects such as hot flushes and genital atrophy; moreover, there is a high rate of endometriosis recurrence (Garai et al. 2006). Therefore, a novel therapeutic drug for satisfactory treatment of endometriosis is needed.

Atorvastatin is a lipid-lowering drug belonging to the statin family of medications (Lau et al. 2006a, b). It is used in the prevention and management of cardiovascular diseases, based on its actions as an inhibitor of HMG-CoA reductase, which is required for cholesterol production (Lau et al. 2006a, b). The anti-angiogenesis and -inflammatory effects of high-dose atorvastatin (Park et al. 2002; Vincent et al. 2002), and its protective effect on endometriosis via inhibition of vascular growth endothelial factor (VEGF) and angiogenesis, (Oktem et al. 2007), have also been described.

The cyanogenic glycoside amygdalin is found in bitter almonds, apricots, peaches, apples, and plums (Lerner 1981). Among its pharmacological activities are anti-cancer, -diabetic, -inflammatory, -asthmatic and -atherosclerotic effects (Jiagang et al. 2011; Abbas et al. 2013). Simsek et al. (2012) showed that amygdalin modulates the activities of local immune cells. Since these cells are an essential component of endometrial growth and development in endometriosis (Simsek et al. 2012), in this study, we examined the potential protective effects of atorvastatin and amygdalin in Sprague-Dawley rats with experimentally induced endometriosis.

## Materials and methods

### Rats

Female Sprague-Dawley rats (220–240 g) were obtained from the animal house (The first affiliated hospital of Wenzhou Medical University, Wenzhou, 325000, China) and divided into four homogeneous groups. Before the study, the rats had free access to food and water and were housed in cages under a standard light period (12/12 h light/dark). All experimental procedures involving rats were monitored and approved by the first affiliated hospital of Wenzhou Medical University, Wenzhou, 325000, China.

### Experimental endometriosis and groups

Experimental endometriosis was induced in the rats according to a previously described method (Demirel et al. 2014). Briefly, rats were anesthetized with intraperitoneal administration of xylazine (8 mg/kg) and ketamine (60 mg/kg). Rats were immobilized on surgery board, and then, ectopic endometrium was induced for the induction of endometriosis. The four groups used in this study were as follows: group I, sham-operated control (normal rats); group II, untreated endometriosis (control); group III, endometriosis treated with atorvastatin (5 mg/kg) with amygdalin (5 mg/kg); and group IV, endometriosis treated with atorvastatin (10 mg/kg) with amygdalin (10 mg/kg). Atorvastatin (Y0001327, Sigma-Aldrich, Shanghai) and amygdalin (A6005-1G, Sigma-Aldrich, Shanghai) were dissolved in dimethyl sulfoxide. Both drugs were orally administered to the rats for 21 consecutive weeks.

### Determination of antioxidant markers

Transplant tissues were homogenized and centrifuged, and the supernatant was used for determination of lipid peroxidation [malondialdehyde (MDA) content]. Briefly, the MDA content was measured as an index of lipid peroxidation in the tissue homogenate by measuring thiobarbituric acid reactive species (TBARS). The resultant final product was measured the absorbance at 534 nm. Reduced glutathione (GSH) levels in the homogenate were determined based on Ellman’s reaction. The final product was measured by the absorbance at 412 nm. Superoxide dismutase (SOD) activity was determined by the addition of 0.1 ml of tissue homogenate, 1.2 ml of sodium phosphate buffer, 0.3 ml of nitro blue tetrazolium and 0.2 ml of NADH. The absorbance was measured at 560 nm. Catalase activity was determined by the addition of 500 µl of phosphate buffer, 500 µl of tissue homogenate, 500 µl of H_2_O_2_, and 500 µl of TiOSO_4_ to the reaction tube. The absorbance was measured at 420 nm. Glutathione peroxidase (Gpx) activity was determined in tissue homogenate by measuring the absorbance at 340 nm (Arutyunyan et al. 2016; Jordão et al. 2004; Erden-Inal et al. 2002).

### Determination of inflammatory markers

Tumor necrosis factor-α (TNF-α), interleukin-6 (IL-6), MMP-2 and MMP-9 levels in the peritoneal fluid were determined (Ziamajidi et al. 2017; Brower et al. 2007).

### RT-PCR

Total RNA was isolated from tissue homogenate and converted into cDNA using oligo (dT) primers. The cDNA was used for qRT-PCR using primers specific for TNF-α, IL-6, MMP-2, and MMP-9 and GAPDH acted as the internal control. Relative expression ratios were determined according to Bernal et al. (2005). Primers specific to the genes encoding the above-listed markers were used in this study and are shown in Table 1.

**Table 1:** List of RT-PCR primers used for the amplification of TNF-α, IL-6, MMP-2 and MMP-9

S. no	Gene name	Forward	Reverse
1	TNF-α	5′-CCCAGACCCTCACACTCAGAT-3′	5′-TTG TCC CTTGAA GAG AAC CTG-3′
2	IL-6	5′-AAGTTTCTCTCCGCAAGATAC TTCCAGCCA-3′	5′-AGG CAAATTTCCTGGTTATATCCA GTTT-3′
3	MMP-2	5′-AGGATCATTGGCTACACACC-3′	5′-AGCTGTCATAGGATGTGCCC-3′
4	MMP-9	5′-CGCAGACATCGTCATCCAGT-3′	5′-GGATTGGCCTTGGAAGATGA-3′
5	GAPDH	5′-TCCCTCAAGATTGTCAGCAA-3′	5′-AGATCCACAACGGATACATT-3′

### Histopathology

At the end of the treatment, transplant tissues were excised, perfused with normal saline, and then fixed in neutral formalin (10%) for 24 h. Tissue dehydration was carried out with graded alcohols, and then tissues were embedded in paraffin. Paraffin-embedded tissues were cut into 4–5-µm sections with a rotary microtome (Leica RM2255, Shanghai China), and hematoxylin and eosin (H&E) was used to stain (Althnaian et al. 2013).

### Statistical analysis

The data are reported as means and standard deviation. One-way ANOVA (SPSS 17, IBM SPSS Statistics, Hong Kong) was used for statistical analysis of the data, and a post hoc Tukey’s test was applied for multiple comparisons. A P-value < 0.05 was considered to indicate statistical significance.

## Results

**Fig. 1:**
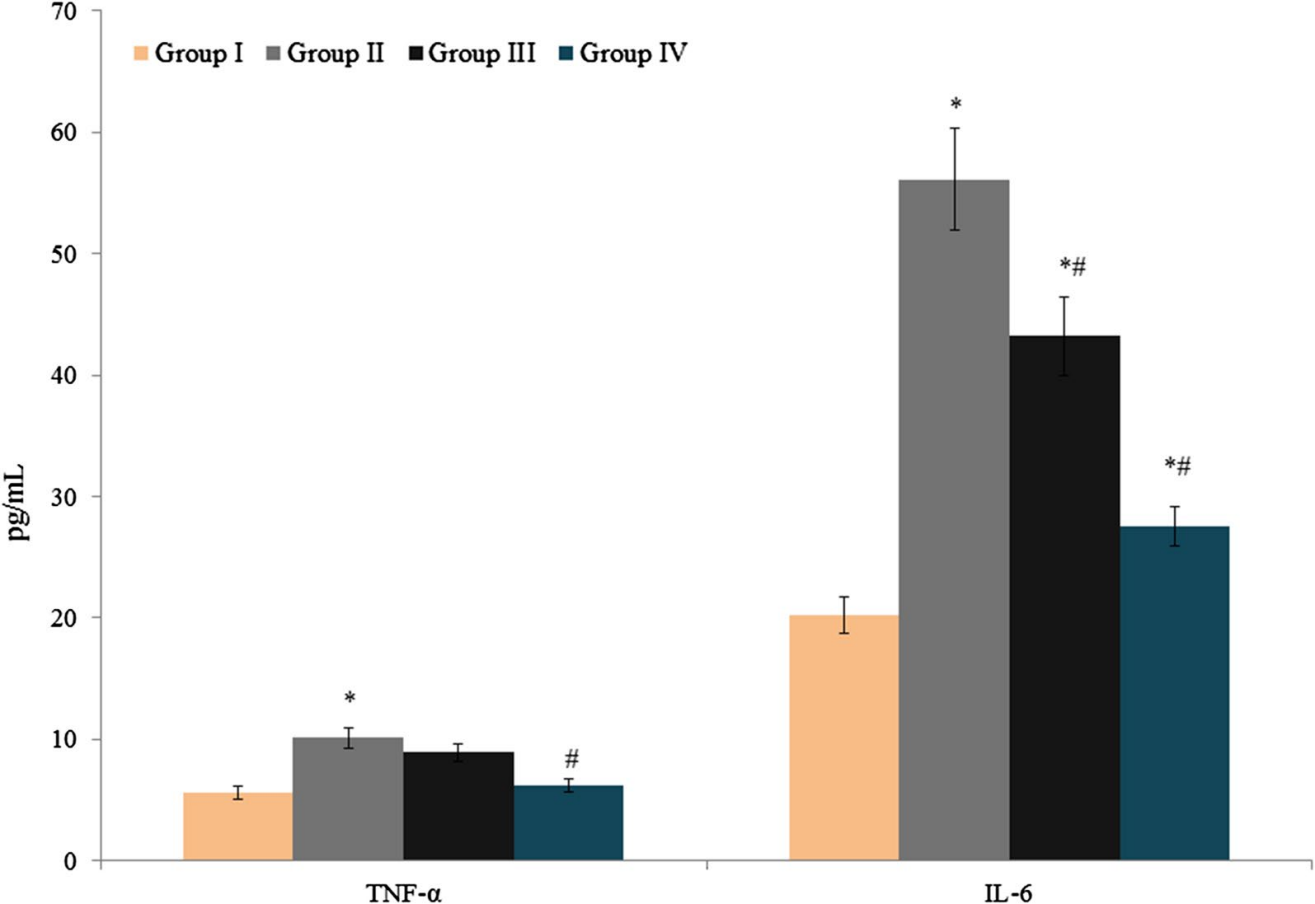
Effect of atorvastatin and amygdalin on tumor necrosis factor-alpha (TNF-α) and interleukin-6 (IL-6) levels in the peritoneal fluid of Sprague-Dawley rats with experimentally induced endometriosis. Values are expressed in pg/ml. *P < 0.05 vs. group I (sham-operated rats) and ^#^*P *< 0.05 vs. group II (non-treated endometriosis control rats)

**Fig. 2:**
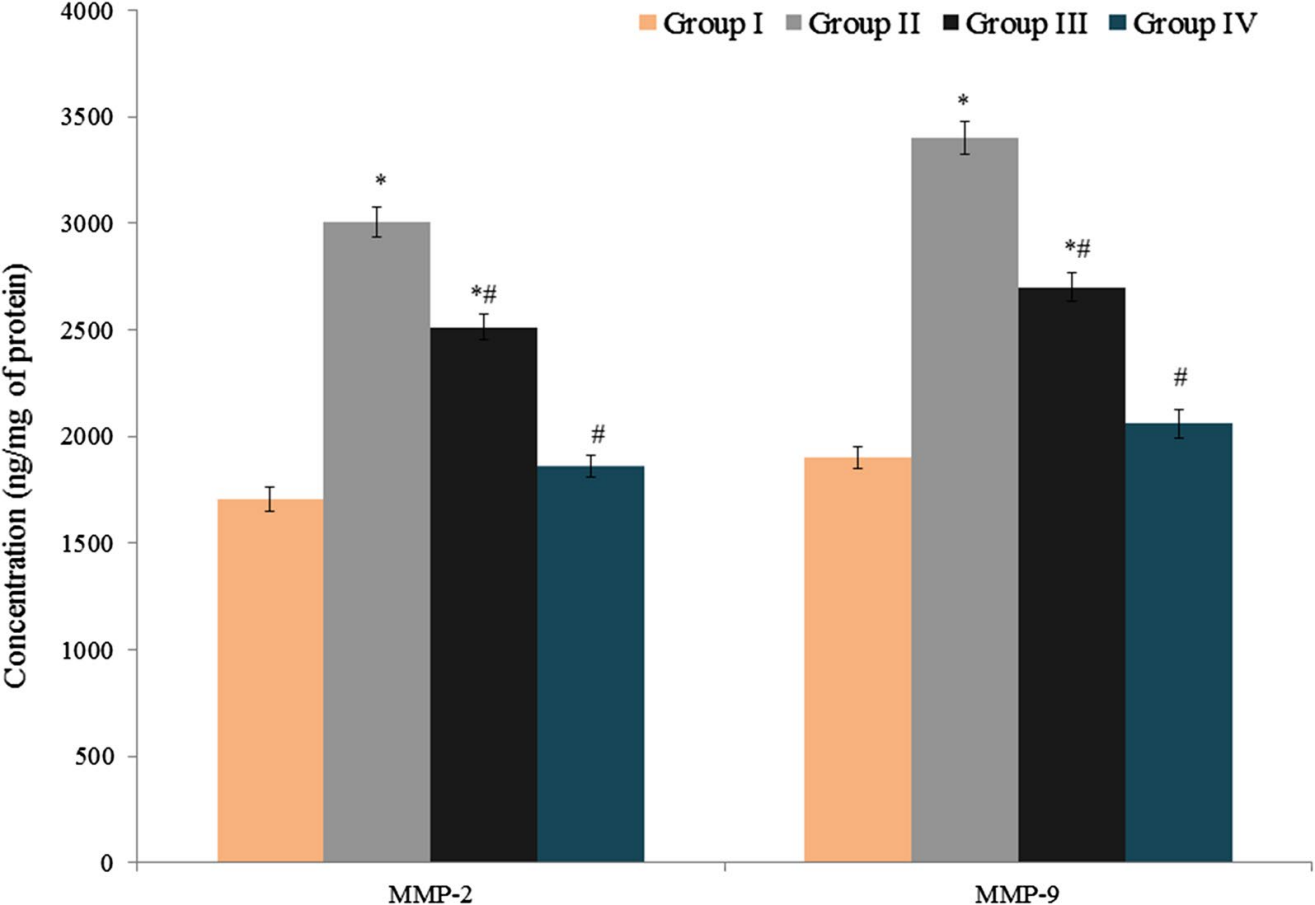
Effect of atorvastatin and amygdalin on matrix metalloproteinase-2 (MMP-2) and MMP-9 in the peritoneal fluid of Sprague-Dawley rats with experimentally induced endometriosis. Values are expressed in ng/mg of protein. *P < 0.05 vs. group I (sham-operated rats) and ^#^*P *< 0.05 vs. group II (non-treated endometriosis control rats)

TNF-α and IL-6 levels were substantially increased (10.1 and 56.1 pg/ml, respectively) in control rats (Fig. 1, *P *< 0.041). Treatment of group III and IV rats with atorvastatin and amygdalin significantly (*P *< 0.046) reduced TNF-α (11.9% and 38.6%, respectively) and IL-6 (23% and 51%) levels (Fig. 1). MMP-9 and MMP-2 levels increased substantially (3400.8 and 3005.6 ng/mg protein, respectively) in control rats with endometriosis (Fig. 2, *P *< 0.033) compared to the sham-operated control, whereas in groups III and IV, combined atorvastatin and amygdalin treatment significantly reduced MMP-2 levels, to 16.4% and 38.1%, and MMP-9 levels, to 20.6% and 39.4% (Fig. 2, *P *< 0.027).

**Fig. 3:**
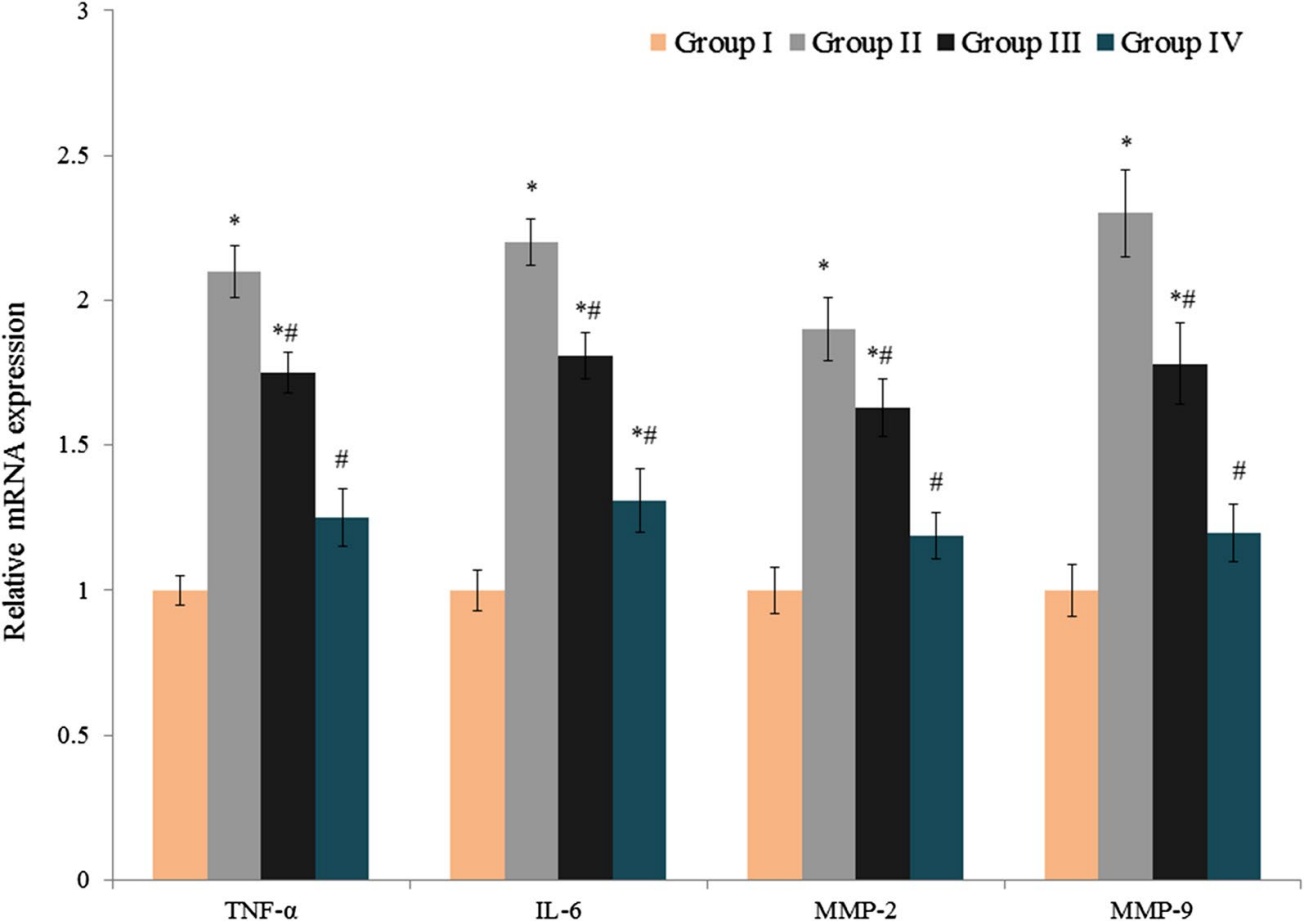
Effect of atorvastatin and amygdalin on the mRNA expression of TNF-α, IL-6, MMP-2 and MMP-9 in the transplant salvage tissue of Sprague-Dawley rats with experimentally induced endometriosis. Values are expressed as fold changes. *P < 0.05 vs. group I (sham-operated rats) and ^#^*P *< 0.05 vs. group II (non-treated endometriosis control rats)

As shown in Fig. 3, TNF-α, IL-6, MMP-2, and MMP-9 mRNA levels were increased substantially in the control transplant salvage tissues (1.1-, 1.2-, 0.9- and 1.3-fold respectively, *P *< 0.034). Treatment of group III and IV rats with atorvastatin and amygdalin significantly reduced TNF-α (0.17 and 0.4 fold, respectively) and IL-6 (0.18- and 0.38-fold) mRNA levels, as well as MMP-2 (0.14- and 0.37-fold) and MMP-9 (0.23- and 0.48-fold) mRNA levels (Fig. 3, *P *< 0.039).

**Fig. 4:**
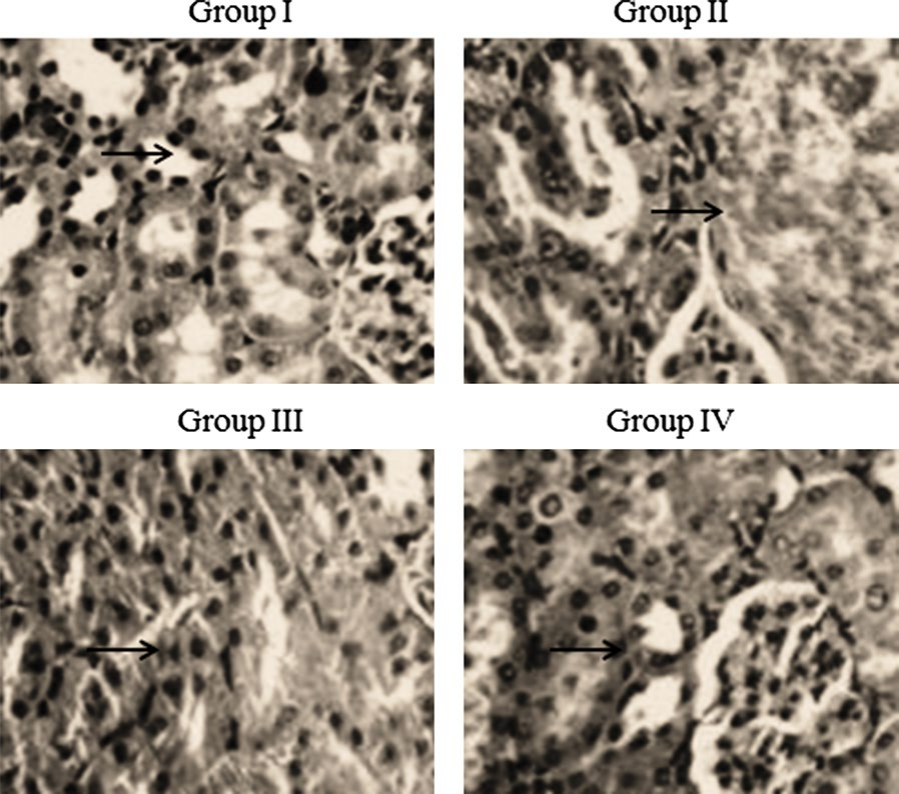
Effect of atorvastatin and amygdalin on the cellular architecture of transplant salvage tissue of Sprague-Dawley rats with experimentally induced endometriosis. Magnification: ×100

The MDA content, measured as the end-product of lipid peroxidation, increased to higher levels in control rats (55.2 nmol/g) whereas in group III and IV rats treated with atorvastatin and amygdalin, the MDA content was significantly reduced (22% and 54%) (Table 2, *P *< 0.026). GSH levels were substantially reduced in control rats (24.6 mg/g), but they increased significantly in group III and IV rats in response to atorvastatin and amygdalin treatment (75% and 193%, respectively, Table 2, *P *< 0.043). The decrease in SOD activity in the control group contrasted with the significantly increased activity in groups III and IV (80.9% and 223.8%, respectively, Table 2, *P *< 0.044). Catalase activity was also substantially reduced in control rats, whereas in groups III and IV, treated with atorvastatin and amygdalin, activity was significantly increased (58.9% and 146.4%, respectively, Table 2, *P *< 0.036). The low level of Gpx activity in control rats contrasted with the significant response to treatment of atorvastatin and amygdalin in groups III and IV (104.5% and 259%, respectively, Table 2, *P *< 0.041). The histopathological analysis showed an increased stromal vessel density and well-preserved epithelium in sham-operated rats, while leukocyte infiltration and fibrosis were seen in control rats. However, these pathological features were significantly reduced in rats treated with atorvastatin and amygdalin (Fig. 4).

**Table 2:** Effect of atorvastatin and amygdalin on lipid peroxidation and antioxidant markers in experimental endometriosis induced Sprague-Dawley rats

Parameters	Group I	Group II	Group III	Group IV
MDA (nmol/g)	21.3 ± 1.7	55.2 ± 3.1*	43.2 ± 2.2*^#^	25.6 ± 2.5^#^
GSH (mg/g)	83.4 ± 6.5	24.6 ± 1.5*	43.1 ± 3.1*^#^	72.2 ± 5.7^#^
SOD (U/mg)	7.8 ± 0.34	2.1 ± 0.12*	3.8 ± 0.12*^#^	6.8 ± 0.17^#^
Catalase (U/g)	15.1 ± 1.5	5.6 ± 0.25*	8.9 ± 0.2*^#^	13.8 ± 0.8^#^
Gpx (mg/protein)	0.91 ± 0.007	0.22 ± 0.005*	0.45 ± 0.005*^#^	0.79 ± 0.005*^#^

* *P* < 0.05 vs. group I

^#^*P* < 0.05 vs. group II

## Discussion

This study evaluated the protective effects of atorvastatin and amygdalin against experimental endometriosis induced in Sprague-Dawley rats. The anti-angiogenesis and anti-inflammatory effects of high-dose atorvastatin, via inhibition of HMG-CoA reductase (Park et al. 2002; Vincent et al. 2002), have also been demonstrated in endometriosis, in which both VEGF and angiogenesis have been implicated (Oktem et al. 2007). Amygdalin, a component of bitter almonds, stone fruits such as apricots, peaches and plums, and the seeds of apples (Lerner 1981), has several pharmacological activities, including anti-cancer, -diabetic, -inflammatory, -asthmatic and -atherosclerotic effects (Jiagang et al. 2011; Abbas et al. 2013), as well as effects on local immune cells (Simsek et al. 2012).

Oxidative stress plays a crucial role in the pathophysiology of endometriosis. Cytokines released from macrophages increase cellular redox status (Bedaiwy and Falcone 2003), as evidenced by the higher Gpx and SOD activities in endometriosis versus normal intact endometrium (Oner-Iyidogan et al. 2004). In our study, combined supplementation with atorvastatin and amygdalin considerably reduced both cytokine levels and increased the Gpx, SOD and CAT activities.

Cell proliferation, invasion, and migration are well regulated by MMPs (Sternlicht and Werb 2001). Zhou and Nothnick (2005) reported that increased levels of MMPs in endometriosis contribute to disease development. MMP-2 and MMP-9 levels were shown to be higher in patients with endometriosis than in healthy controls (Chung et al. 2002; Huang et al. 2004; Nguyen et al. 2016). They are also higher in the peritoneal fluid of mice and humans (Zong et al. 1999; Gottschalk et al. 2000). Our results showed that the combination of atorvastatin and amygdalin significantly reduced MMP-2 and MMP-9 mRNA expression, which suggests that both drugs act at the transcriptional level. The increased stromal vessel density and well-preserved epithelium in sham rats contrasted with the leukocyte infiltration and fibrosis that characterized the endometriosis group. However, treatment with atorvastatin and amygdalin significantly ameliorated these pathological features. This finding is consistent with a report showing that amygdalin treatment considerably reduces the pathological score in endometriosis (Brown and Farquhar 2014). Our study suggests that combined treatment with atorvastatin and amygdalin considerably attenuates endometriosis. However, the molecular mechanism underlying the action of atorvastatin and amygdalin action in endometriosis remains to be investigated in detail.
